# Two-Step Image Registration for Dual-Layer Flat-Panel Detectors

**DOI:** 10.3390/diagnostics14232742

**Published:** 2024-12-05

**Authors:** Dong Sik Kim, Dayeon Lee

**Affiliations:** Department of Electronics Engineering, Hankuk University of Foreign Studies, Gyeonggi-do, Yongin-si 17035, Republic of Korea; yeooon@hufs.ac.kr

**Keywords:** convex combination image, detective quantum efficiency, dual-layer flat-panel detector (DFD), image registration

## Abstract

Background: For a single exposure in radiography, a dual-layer flat-panel detector (DFD) can provide spectral images and efficiently utilize the transmitted X-ray photons to improve the detective quantum efficiency (DQE) performance. In this paper, to acquire high DQE performance, we present a registration method for X-ray images acquired from a DFD, considering only spatial translations and scale factors. The conventional registration methods have inconsistent estimate accuracies depending on the captured object scene, even when using entire pixels, and have deteriorated frequency performance because of the interpolation method employed. Methods: The proposed method consists of two steps; the first step is conducting a spatial translation according to the Fourier shift theorem with a subpixel registration, and the second step is conducting a scale transformation using cubic interpolation to process the X-ray projections. To estimate the subpixel spatial translation, a maximum-amplitude method using a small portion of the slant-edge phantom is used. Results: The performance of the proposed two-step method is first theoretically analyzed and then observed by conducting extensive experiments and measuring the noise power spectrum and DQE. An example for registering chest images is also shown. For a DFD, the proposed method shows a better registration result than the conventional one-step registration. The DQE improvement was more than 56% under RQA 9 compared to the single flat-panel detector case. Conclusions: The proposed two-step registration method can efficiently provide aligned image pairs from the DFD to improve the DQE performance at low doses and, thus, increase the accuracy of clinical diagnosis.

## 1. Introduction

Dual-layer flat-panel detectors (DFDs) enable single-exposure spectral imaging based on energy-selective imaging from copper filtering or beam hardening [1,2,3]. These detectors have a variety of applications, such as in bone and tissue separations [1], material decomposition [4], and bone mineral density estimation [5]. They can also improve the contrast-to-noise ratio and detective quantum efficiency (DQE) [6,7,8]. Engel et al. [7] showed an increased DQE from DFDs compared to a single-layer detector case having an added thickness of the scintillator layers of DFD. Kim [8] showed that convex combination of upper and lower images acquired from DFD produces higher DQE and reduces noise power spectrum (NPS) [9]. Su et al. [10] obtained super-resolution images based on a DFD. Dual-energy cone-beam computed tomography is a suitable field for the application of DFD [4,5,6].

**Figure 1:**
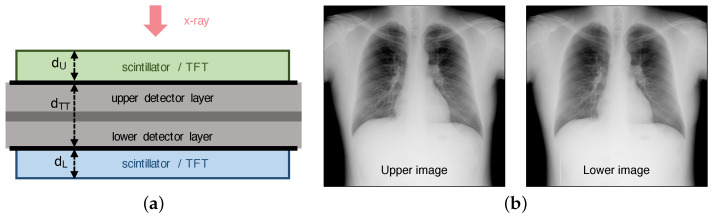
Structure of the dual-layer flat-panel detector (DFD) and acquired images (DRTECH Co., Ltd., Seongnam, Republic of Korea, www.drtech.com). (**a**) Two flat-panel detector layers are attached as close as possible. Conventionally, the upper and lower layers can have the same direction as the incident X-ray. In this example, the lower layer is inverted. The upper layer can also be inverted [8]. $d_{\mathrm{TT}}$ is the distance between TFT layers, and $d_{U}$ and $d_{L}$ are the thicknesses of the upper and lower scintillator layers, respectively. (**b**) An example of chest images acquired from a DFD. The pixel pitch is 0.14 mm, and the image size is 3072×3072 pixels, where $d_{\mathrm{TT}}=1.1$ mm and $d_{U}=d_{L}=0.5$ mm.

As shown in Figure 1, the structure of a DFD consists of two layers, where the upper and lower detector layers are attached as close as possible [3,11]. The lower layer usually absorbs relatively high-energy X-ray photons because of the beam hardening compared to the upper layer case. The intermediate layer between the upper and lower layers can prevent the mutual transmission of light photons and can contain a spectral filter for X-rays [1,8,12]. The DFDs introduced in the literature are summarized in Table 1, where indirect conversion flat-panel detectors with CsI(Tl)-scintillator layers and pixel pitches of 0.140 mm and 0.145 mm are used [3,5,6,8,10,11].

When stacking the upper and lower detector layers to construct a DFD, the images acquired from the layers should be registered because misalignments occur between the layers. By using a physical positioning device, the rotational deviation between the layers can be controlled to be as small as possible while stacking the layers. However, physically aligning the detector-element positions of the lower thin-film transistor (TFT) layers with respect to those of the upper layer is not easy [8,11]. Hence, a geometric translocation of the lower layer exists in both horizontal and vertical directions with respect to the upper layer.

To conduct an image registration, geometric transformations are first estimated between the upper and lower images acquired with an object and then are employed to register the images based on interpolation schemes [13]. A variety of image registration methods have been developed for applications such as medical imaging, general imaging, remote sensing, and measurement [14,15,16,17,18,19]. Recent studies on the registration of detector layers for DFDs are summarized as follows. Shi et al. [3] registered the lower image using an affine transform accounting for translation, rotation, and scale based on interpolation with the IsoCal phantom [20]. Kim [8] and Lee and Kim [11] estimated the translation parameters based on a slant-edge phantom, which was used for measuring the modulation transfer function (MTF), and conducted transforms with those translation parameters. Wang et al. [5] also aligned the lower image based on an interpolation. However, the employed registrations and transforms were usually based on interpolation schemes and, thus, caused registration errors because of the amplitude and phase distortions of interpolation. Note that the traditional intensity-based and feature-based registration approaches [14,21], which use natural scenes, produce large registration errors and, thus, are not appropriate for the registration purpose of DFDs.

When registering the upper and lower images acquired from a DFD, the following two items should be considered:

- Spatial translation while stacking the layers;
- Scale due to the X-ray projection.

**Figure 2:**
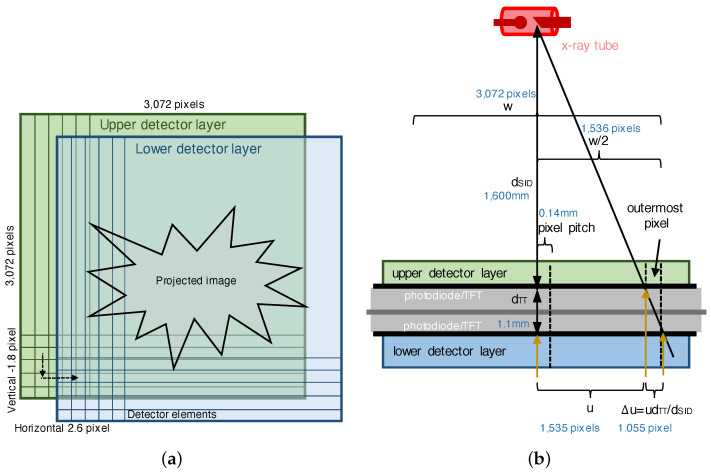
Misalignment example for translation and scale parameters in DFD. (**a**) Translation misalignment while stacking. The lower layer is translated with respect to the upper layer by (2.6, −1.8) pixels to be registered. We assume that the stacked angle between the layers is approximately zero. (**b**) Scale misalignment due to X-ray projection. For $d_{\mathrm{SID}}=1600$ mm and $d_{\mathrm{TT}}=1.1$ mm, the scale factor is 1.0006875, and the outermost pixel, which has its start location at u=1535 pixels from the center, consequently has an image deviation of 1.055 pixel.

The first item is concerned with the registration of the spatial horizontal and vertical translations, as shown in Figure 2a. In a DFD, image registration is a simple process of finding the misaligned spatial translations and using these to transform the lower image. Here, a subpixel registration method [16,22,23,24,25,26,27,28,29,30] is required to accurately find the spatial translation. Subpixel registration is not only important for obtaining aligned image pairs but also for checking the uniform pixel alignment of the stacked detector layers [10]. If the translation parameter of a DFD is accurately estimated, then, using the parameter, the translation of the images acquired from the same DFD can be performed every time to acquire registered image pairs. Hence, there is no need to perform separate image registration in the application of an image processing method. The second item is concerned with a transform of the image scale change due to the X-ray projection, as shown in Figure 2b. Because X-rays generated in the form of a point source in an X-ray tube are projected onto the DFD, the X-ray image obtained from the lower layer is more magnified than the image obtained from the upper layer.

In this paper, a two-step image registration method for a DFD is introduced. Conventional one-step registration methods have errors depending on objects in the X-ray images acquired from the upper and lower detector layers [14]. For spectral purposes, these errors can be ignored. However, the purpose of the considered DFD is to improve DQE and obtain high-quality images even at low doses. Therefore, for this purpose, very precise alignment is required to obtain a performance that is close to a theoretically achievable performance. The proposed method for high-precision image registration for DFDs consists of two steps; the first step is conducting a spatial translation according to the Fourier shift theorem [31] with a subpixel registration, and the second step is conducting a scale transformation using cubic interpolation to process the X-ray projections. To conduct an accurate subpixel registration, we employed a method based on the notion of maximum amplitude, where a conventional slant-edge phantom is used as a fiducial mark [11]. This maximum-amplitude method can provide high-precision spatial translation compared to the methods that use the entire pixels of natural scenes [16,22,23,25,26,27,28,30]. The proposed two-step method can achieve more accurate image registration than the conventional one-step case, especially for DFD applications.

This paper is organized in the following way. In Section 2, we first describe the registration of images acquired from a DFD. We then propose a two-step registration method for a DFD. Theoretical analysis of the proposed method is also conducted to observe the registration performance. To evaluate the registration accuracies experimentally, extensive experiments using X-ray images acquired from the DFD are presented in Section 3 with discussions. The conclusion is then stated in the last section.

## 2. Methods

In this section, we first comparatively discuss subpixel registration methods for the images acquired from a DFD. We then introduce a two-step registration method that takes into account the magnified image due to the X-ray projection.

### 2.1. Two-Step Registration for the Dual-Layer Flat-Panel Detector

In order to obtain an aligned image pair from the DFD, the lower image acquired from the lower detector layer should be accurately aligned with respect to the upper layer. We first consider the translation of horizontal and vertical directions, as shown in Figure 2a, where the lower detector is translated by (−2.6, 1.8) pixels as an example. We can transform the lower image by using the translation parameter based on the Fourier shift theorem or interpolation schemes, such as linear or cubic interpolations. Here, the translation parameter can be estimated from a subpixel registration method [22]. Conventional intensity-based subpixel registration algorithms generally use the entirety of pixels of natural scenes and, thus, are not suitable for finding such a fine translation of subpixel resolutions for a DFD. Note that if the entire pixels of a typical scene are used for image registration, pixels that are not suitable for alignment are also emphasized and may interfere with precise alignment.

**Figure 3:**
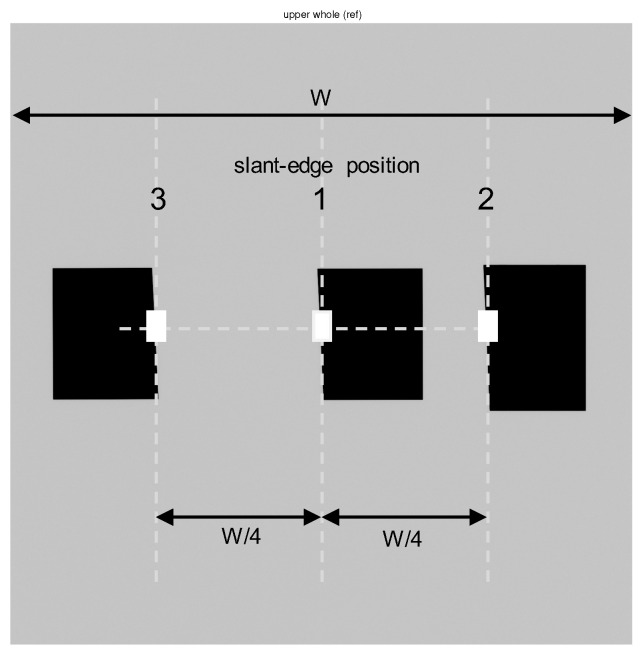
Example images of three slant-edge phantoms for measuring horizontal MTF curves at three different locations.

To increase the accuracy of subpixel registration for a given small area, we should design a complicated phantom with special patterns as a fiducial mark. However, designing a phantom with such a special pattern is difficult for radiography detector applications. Instead of using such a special phantom, Kim [8] used the conventional slant-edge phantom and maximized the DQE value to obtain a registered convex combination image. Lee and Kim [11] recently conducted a subpixel registration based on a necessary condition on maximizing an amplitude response and extended this notion to developing a high-precision measurement algorithm, where a cyclic-coordinate optimization based on the maximum amplitude is conducted. Note that this method can find local subpixel translations using a small portion of the slant edge as Positions 1, 2, and 3 in Figure 3.

Because the X-ray generated in the form of a point source from the X-ray tube is projected onto the DFD, the X-ray image acquired from the lower layer is more enlarged than the image obtained from the upper layer. As shown in Figure 2b, letting $d_{\mathrm{SID}}$ denote the source-to-image distance (SID), a horizontal pixel location of *u* from the aligned center pixel produces the deviation $\Delta u:=ud_{\mathrm{TT}}/d_{\mathrm{SID}}$ pixels in the lower layer with respect to the upper layer. In other words, compared to the upper image, the lower image is enlarged by a scale factor of $1+d_{\mathrm{TT}}/d_{\mathrm{SID}}$, which is derived from the distance $d_{\mathrm{TT}}$, as shown in Figure 2b. To minimize the deviation Δu due to the projection, we need to minimize the distance $d_{\mathrm{TT}}$ between the TFT layers of the upper and lower detector layers. Several examples of the distance are shown in Table 1. We can compensate for the enlarged lower image using the scale factor and an interpolation scheme to conduct a high-precision image registration considering the projection. We can also obtain the scale factor experimentally by measuring pixel deviations from the three slant-edge phantoms of Figure 3.

By using the estimated translation and scale parameters, we can conduct an image transformation based on an interpolation [13]. However, the employed interpolation usually deteriorates the frequency responses of the transformed image. To alleviate the deterioration, we propose a two-step method based on the Fourier shift and scaling with an interpolation. The proposed method for a DFD is summarized as follows.

Two-Step Registration for the Dual-Layer Flat-Panel Detector:

(0) Find the translation of the lower image based on a subpixel registration; calculate the scale factor for a given SID.
(1) Translate the lower image using the translation estimate based on the Fourier shift theorem.
(2) Transform the lower image using the scale factor based on a cubic interpolation.

Note that the proposed method is composed of two steps: translation based on the Fourier shift theorem and scaling based on a cubic interpolation. Instead of these two steps, we can consider a one-step translation with the translation and scale parameters based on an appropriate interpolation, such as linear or cubic schemes. However, this single translation can reduce the MTF response and distort the phase response because of the employed interpolation scheme [13]. On the other hand, the proposed two-step method can alleviate the degradation problem from the interpolation-based transformation approach.

### 2.2. Modulation Transfer Function of the Convex Combination Image

We analyze the registration performance of the proposed registration method by observing the MTF of a convex combination image.

For a pixel position u, let p[u] denote a convex combination [5,8] of the acquired upper and lower images $p_{U}$ and $p_{L}$, respectively, as

(1) $$\begin{array}{c} p\left(u\right):=\lambda p_{U}\left(u\right)+\left(1-\lambda \right)p_{L}\left(u\right), \end{array}$$

with a combination coefficient of λ such that 0≤λ≤1. Here, optimal coefficients of λ can be found in terms of maximizing the NPS or DQE performance [8]. The mean of Equation (1) is given as $\mu :=\lambda \mu_{U}+\left(1-\lambda \right)\mu_{L}$, where $\mu_{U}$ and $\mu_{L}$ are the means of the upper and lower images, respectively. The frequency performance of imaging systems can be evaluated by measuring the detector MTF, which is the amplitude response acquired from the Fourier transform of the impulse response or the point spread function. We first measure directional MTF curves from the convex combination image *p* based on a method described in the IEC standard [32]. Here, to avoid overlaps of aliases and increase measurement accuracies [33], the oversampled impulse response is calculated from the derivative of the oversampled step response, which is acquired from upper and lower X-ray images using a tungsten slant-edge phantom.

At Position 1 of Figure 3, a directional translation of $s_{0}$ is estimated based on a subpixel registration method. This step corresponds to Step (0) in preparation for the proposed registration. By multiplying $e^{-j\omega s}$ in the discrete-time Fourier transform (DTFT) domain, the lower image is aligned based on the Fourier shift theorem [11,31]. This step corresponds to Step (1) of the proposed registration. The directional MTF of the convex combination image, which is denoted as *T*, then satisfies the following relationship:

(2) $$\begin{array}{c} T\left(\omega ,s\right):=\frac{\sqrt{a_{1}\left(\omega \right)\cos\left[\omega (s_{0}-s)\right]+a_{2}\left(\omega \right)}}{\mu }, \end{array}$$

where ω is the normalized radian frequency given as $\omega :=2\pi f/f_{s}$ and |ω|≤π in the DTFT domain. In (2), $a_{1}$ and $a_{2}$ are defined as $a_{1}\left(\omega \right):=2\lambda \left(1-\lambda \right)\mu_{U}\mu_{L}T_{U}\left(\omega \right)T_{L}\left(\omega \right)$ and $a_{2}\left(\omega \right):={\left[\lambda \mu_{U}T_{U}\left(\omega \right)\right]}^{2}+{\left[\left(1-\lambda \right)\mu_{L}T_{L}\left(\omega \right)\right]}^{2}$, respectively, where $T_{U}$ and $T_{L}$ denote the upper and lower MTF curves, respectively [8]. If the estimate *s* from a subpixel registration method is equal to $s=s_{0}$, then the maximum MTF of Equation (2) can be rewritten as

(3) $$\begin{array}{c} T\left(\omega ,s_{0}\right)=\frac{\lambda \mu_{U}T_{U}\left(\omega \right)+\left(1-\lambda \right)\mu_{L}T_{L}\left(\omega \right)}{\mu }, \end{array}$$

which is a convex combination and, thus, is between those of the upper and lower layers. Note that a relationship of $T\left(\omega ,s\right)\leq T\left(\omega ,s_{0}\right)$ holds. Hence, the lower image should be aligned with the true $s_{0}$ to maximize the MTF performance.

If we conduct the translation based on an interpolation scheme instead of the Fourier shift theorem, then the MTF of Equation (2) is reduced because of the interpolation. Let Ψ denote such a reduced MTF. The MTF from the interpolation is then given as

(4) $$\begin{array}{c} \Psi \left(\omega ,s\right):=\frac{\sqrt{b_{1}\left(\omega ,s\right)\cos\left[\omega s_{0}+\beta \left(\omega ,s\right)\right]+b_{2}\left(\omega ,s\right)}}{\mu }, \end{array}$$

where the constants $b_{1}$ and $b_{2}$ are defined as

(5) $$\begin{array}{c} b_{1}\left(\omega ,s\right):=2\lambda \left(1-\lambda \right)\mu_{U}\mu_{L}\alpha \left(\omega ,s\right)T_{U}\left(\omega \right)T_{L}\left(\omega \right) \end{array}$$

and

(6) $$\begin{array}{c} b_{2}\left(\omega ,s\right):={\left[\lambda \mu_{U}T_{U}\left(\omega \right)\right]}^{2}+{\left[\left(1-\lambda \right)\mu_{L}\alpha \left(\omega ,s\right)T_{L}\left(\omega \right)\right]}^{2}, \end{array}$$

respectively. In Equations (4)–(6), α and β are the amplitude and phase responses, respectively, from the translation with interpolation.

We now observe the amplitude and phase responses of the interpolation-based translation to observe the reduced MTF of Equation (4). Let $\alpha_{1}$ denote the amplitude response of a translation of *s* to the right direction based on linear interpolation (B-spline of degree 1 [34]). $\alpha_{1}$ is then given as

(7) $$\begin{array}{c} \alpha_{1}\left(\omega ,s\right):=\sqrt{1-2|s|(1-\left|s|\right)\left[1-\cos(\omega )\right]}, \end{array}$$

for |s|≤1, and is periodic with a fundamental period of 1 [11]. Here, the relationship $0\leq \alpha_{1}\leq 1$ is satisfied. In a similar manner, the amplitude response of the translation $\alpha_{3}$, which is based on a cubic interpolation (Catmull–Rom spline [35]), is given as

(8) $$\begin{array}{c} \alpha_{3}\left(\omega ,s\right):=\left[1+|s|(1-\left|s|\right)\left[1-\cos(\omega )\right]\right]\alpha_{1}\left(\omega ,s\right). \end{array}$$

Because a relationship of $\alpha_{1}\leq \alpha_{3}\leq 1$ holds, we can alleviate the amplitude reduction problem by using cubic interpolation instead of linear interpolation. In (5) and (6), α can be $\alpha_{1}$ or $\alpha_{3}$ for the linear or cubic interpolation, respectively. Furthermore, the phase responses of both linear and cubic interpolations are given as

(9) $$\begin{array}{c} \beta \left(\omega ,s\right):=-\arctan\left[\frac{s\sin(\omega )}{1-\left|s\right|\left[1-\cos(\omega )\right]}\right] \end{array}$$

for a translation of *s* [11].

**Figure 4:**
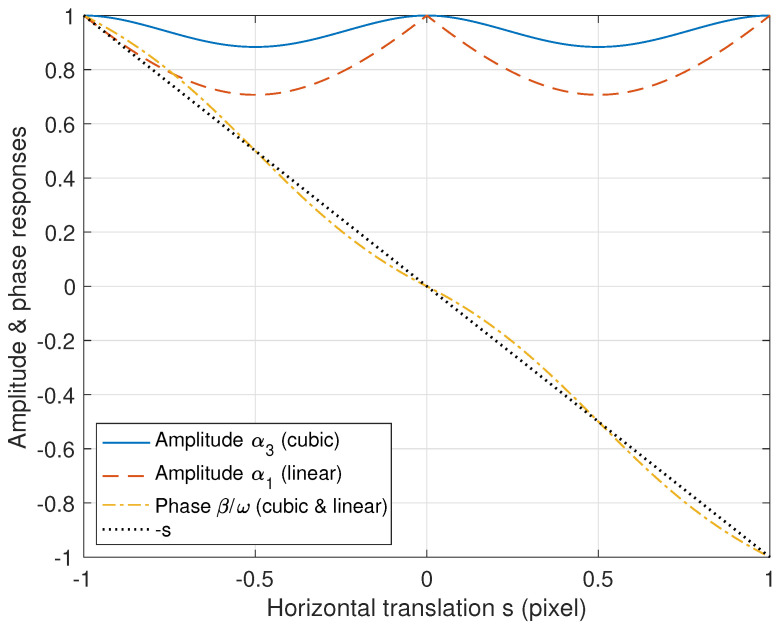
Amplitude and phase responses of the translation with *s* based on linear (B-spline of degree 1 [34]) and cubic (Catmull–Rom spline [35]) interpolations, where ω=π/2.

Examples of the amplitude responses on $\alpha_{1}$ and $\alpha_{3}$ and phase responses on β are illustrated in Figure 4. When the translation *s* is an integer, the amplitude response becomes the largest value, 1, and when the remainder divided by an integer is 0.5, it becomes the smallest value. We can also observe in Figure 4 that the curve near the integer translations in the amplitude response of cubic interpolation is gentler and smoother than the linear interpolation case. Note that Ψ(ω,s)≤T(ω,s) is satisfied, where the equality holds when *s* is an integer. Hence, if the true translation $s_{0}$ is not an integer, then $\Psi \left(\omega ,s_{0}\right)<T\left(\omega ,s_{0}\right)$ holds. Therefore, even if aligned precisely to the true $s_{0}$, the interpolation-based translation does not guarantee the maximum MTF performance.

### 2.3. Noise Power Spectrum and the Detective Quantum Efficiency

We now observe a noise performance of the convex combination. Let $P_{U}$ and $P_{L}$ denote the normalized NPS (NNPS) of the upper and lower layers, respectively [9]. The directional NNPS, which is denoted as *P*, has an approximate relationship:

(10) $$\begin{array}{c} \min_{\lambda }P\left(\omega \right)\approx {\left[\frac{1}{P_{U}\left(\omega \right)}+\frac{1}{P_{L}\left(\omega \right)}\right]}^{-1}, \end{array}$$

which is a harmonic mean of the upper and lower NNPS values [8]. For a given ω, an optimal coefficient $\lambda_{1}\left(\omega \right)$, which yields the Equation (10), can be calculated by using the upper and lower NPS values [8]. From Equation (10), we notice that $\min_{\lambda }P\left(\omega \right)$ is less than both $P_{U}\left(\omega \right)$ and $P_{L}\left(\omega \right)$. In other words, the NNPS of the convex combination can be improved from those of the upper and lower layers if the images are registered.

We next observe and analyze the DQE performance. The DQE value denoted as *Q* can be written as $Q\left(\omega \right):=T^{2}\left(\omega \right)/\Phi P(\omega )$, where Φ is the mean quanta per area. An optimal coefficient of λ, which is denoted as $\lambda_{2}\left(\omega \right)$, for maximizing *Q* can be calculated by using the upper and lower NPS and MTF curves for a fixed ω [8]. If $\lambda =\lambda_{2}\left(\omega \right)$, then we can obtain an algebraic DQE summation asymptotically:

(11) $$\begin{array}{c} \max_{\lambda }Q\left(\omega \right)\approx Q_{U}\left(\omega \right)+Q_{L}\left(\omega \right) \end{array}$$

for a fixed ω, where $Q_{U}$ and $Q_{L}$ are the upper and lower DQE values, respectively [8]. Hence, we can significantly improve the detector DQE performance from the DFD.

### 2.4. Projection and a Scale Translation with Interpolation

**Figure 5:**
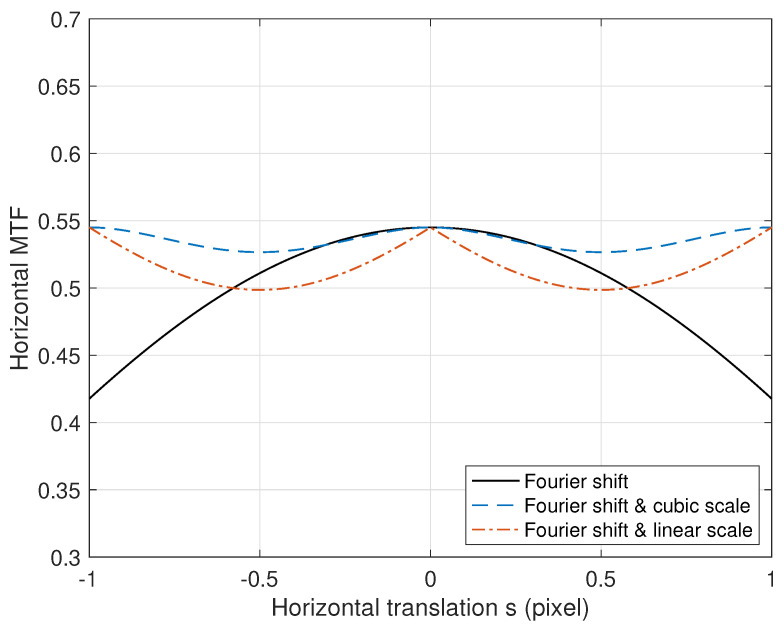
MTF comparison with respect to the shift *s*, where ω=π/2. “Fourier shift” implies the MTF with a misalignment of *s* and given as T(ω,s) with $s_{0}=0$. “Fourier shift & cubic scale” implies the scale transform with the cubic interpolation and shows better MTF performance than when there is no scale transformation. In this case, the MTF value is given as Ψ(ω,s) with $s_{0}=s$ and $\alpha =\alpha_{3}$. “Fourier shift & linear scale” is the result based on linear interpolation.

We now analyze the scale translation of Step (2) of the proposed registration. We assume that the lower image is translated based on the Fourier shift theorem, and hence, the true shift satisfies $s_{0}=0$. As shown in Figure 3, the pixel location *u* from the center produces a projection deviation of Δu. Because of this projection, the shift between the upper and lower images is given as s=Δu and decreases the MTF value as T(ω,s) of Equation (2) with $s_{0}=0$. We can observe from “Fourier shift” in Figure 5 that the MTF value with a misalignment of *s* is reduced as |s| increases.

To alleviate the deviation problem due to the enlarged lower image, we conduct a transformation with the scale factor to the lower image, which is translated based on the Fourier shift theorem. This step corresponds to Step (2) of the proposed registration. The resultant MTF is given as Ψ(ω,s) of Equation (4) with $s_{0}=s$ and is shown in Figure 5. For the linear and cubic interpolation cases, α is $\alpha_{1}$ and $\alpha_{3}$, respectively. The cubic interpolation “Fourier shift & cubic scale” in Figure 5 shows a better MTF performance than the case without scale translation. In other words, even though the interpolation from the scale translation reduces the MTF value, conducting a scale transformation can improve the MTF performance. Therefore, we notice that the proposed two-step approach can yield a better performance than the conventional case of one-step transforms. In other words, if the incident exposure is uniform for a given SID, nearly uniform MTF and DQE performance can be achieved across all pixels in the image.

## 3. Results and Discussion

In this section, we observe the registration performance of the proposed high-precision two-step registration method experimentally for the DFD, which was constructed by stacking upper and lower detector layers, as shown in Figure 1 (DRTECH Co., Ltd., Seongnam, Republic of Korea, www.drtech.com) [8]. Each layer has a CsI(Tl)-scintillator layer with 3072×3072 photodiode pixels controlled by an amorphous indium-gallium-zinc-oxide (a-IGZO) TFT panel. The pixel pitch is 140 μm, yielding a sampling frequency of $f_{s}=7.143$ lp/mm, and the resolution is 16 bits/pixel. The thicknesses of the upper and lower scintillator layers are $d_{U}=0.35$ mm and $d_{L}=0.5$ mm, respectively.

**Figure 6:**
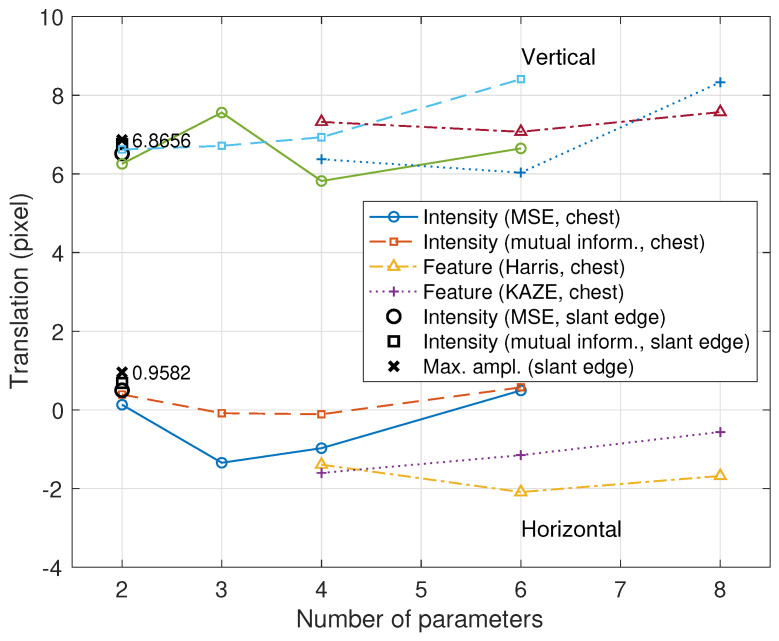
Translation estimate comparison from intensity-based and feature-based image registrations using chest image pairs [36,37,38,39,40]. For the number of parameters, “2” implies horizontal and vertical translations, “3” additionally has rotation, and “4” additionally has scale. “6” implies the affine transform, and “8” implies the projection transform [13]. “Max. ampl. (slant edge)” has a translation of (0.9582,6.8656) using the slant-edge phantom.

For Step (0), several methods were applied to estimate the translation parameters, and the results are illustrated in Figure 6. The conventional registration methods using chest images show erroneous deviations compared to the methods that use the slant-edge phantom as a fiducial mark to estimate only two translation parameters. The maximum-amplitude method yielded a translation estimate of (0.9582,6.8656), which is shown as an accurate estimate in terms of the MTF and DQE in this section. Here, the employed maximum-amplitude method shows a mean absolute error of $0.3021\times 10^{-3}$ compared to $11.46\times 10^{-3}$ from a conventional gradient-based subpixel registration [11]. In Step (1), the lower image was shifted using the translation estimate based on the Fourier shift theorem, and in the following step, Step (2), the shifted image was then transformed by using the scale factor $1+d_{\mathrm{TT}}/d_{\mathrm{SID}}=1.0006875$ with an interpolation method, where $d_{\mathrm{TT}}=1.1$ mm and $d_{\mathrm{SID}}=1600$ mm.

### 3.1. Numerical Performance Observation

**Figure 7:**
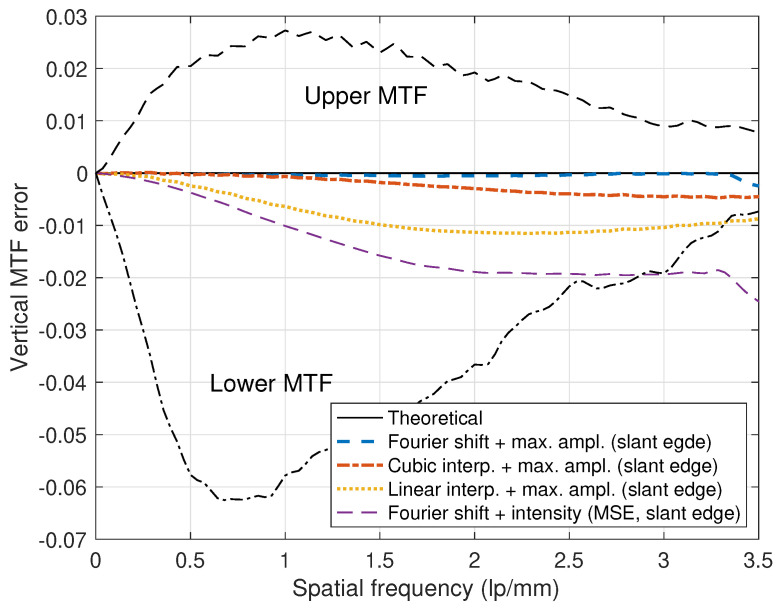
Measured MTF error comparison (the thicknesses of the upper and lower scintillator layers are $d_{U}=d_{L}=0.5$ mm, the X-ray beam quality is RQA 9, the subpixel translation is (0.9582,6.8656) based on the maximum amplitude, and the combination coefficient is λ=0.525). The empirical MTF based on the Fourier shift theorem is between those of the upper and lower detector layers and is close to the theoretical value. The translations based on the cubic and linear interpolations show lower MTF values than the theoretical case because the amplitude reduction and phase delay arise from interpolation. An intensity-based registration for the translation is erroneous by (0.5000, 6.5175), as indicated by “Intensity (MSE, slant edge)” in Figure 6.

In Figure 7, numerical results on measuring directional MTF values are illustrated for the convex combination of the registered images from the proposed registration method. From “Theoretical” and “Trans. (Fourier shift)” in Figure 7, we can observe that the MTF of the convex combination is very close to the theoretical value. As shown in Equation (3), we can observe that the theoretical MTF is between the upper and lower MTF values. If we conduct the translation based on the interpolation scheme, then the MTF is reduced, as mentioned in Equations (7) and (8) (“Cubic interp.+max ampl. (slant edge)” and “Linear interp.+max ampl. (slant edge)” in Figure 7).

If the lower image is not aligned, then the MTF values of relatively high frequencies are usually lower than the theoretical ones. Hence, precisely aligning the lower image is important to ensure good MTF performance even at relatively high frequencies. In the intensity-based or feature-based subpixel registration methods, the estimation accuracy depends on the object of the X-ray image and can be degraded, as shown in Figure 6. For example, in Figure 7, the translation result from the maximum amplitude is close to the theoretical value (“Fourier shift+max ampl. (slant edge)”). However, the shift parameter estimated from an intensity-based subpixel registration method shows lower values than the theoretical case (“Fourier shift+intensity (MSE, slant edge)”).

**Figure 8:**
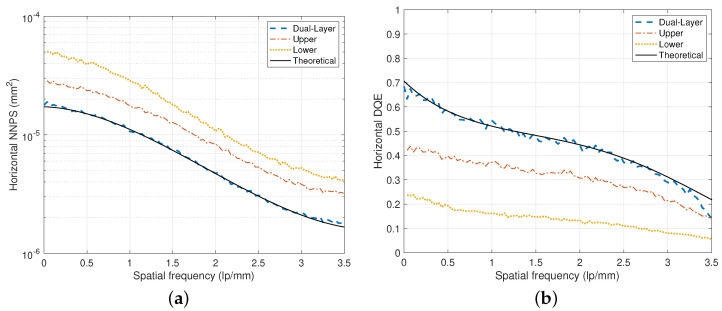
Measured NNPS and DQE of the dual-layer detectors, where $d_{U}=0.20$ mm and $d_{L}=0.5$ mm. The X-ray beam quality is RQA 9, the incident dose is 2.65 μGy, and λ=0.525. (**a**) NNPS. (**b**) DQE.

Directional NNPS measurement examples are illustrated in Figure 8a. Here, we compensated for the NNPS values, which were inflated during the gain correction procedure, by considering the number of white images acquired under an incident exposure for the gain map design [9,41]. We can observe that the NNPS value of the upper layer is less or better than that of the lower layer and the NNPS value of the convex combination is less than those of both layers. Hence, to improve the noise performance, we can use the convex combination of the images from both upper and lower layers for an appropriate combination coefficient of λ. In the experiments of Figure 7 and Figure 8a, optimal values of $\lambda_{1}\left(0\right)$ are used for the combination coefficient.

Under an X-ray beam of RQA 9, DQE experiments are illustrated in Figure 8b for an optimum of $\lambda_{2}\left(0\right)=0.525$, which is equal to $\lambda_{1}\left(0\right)$. As observed in this figure, the DFD shows improved DQE values close to theoretically achievable values, which are derived based on a parametric model [8]. Here, the MTF and NPS curves are modeled by performing third-order polynomial fits. For the experiments of the detector, the optimal curve of $\lambda_{2}\left(\omega \right)$ is approximately constant for all ω [8]. Hence, we can observe from Figure 8b that $Q\approx Q_{U}+Q_{L}$ for overall frequencies, as shown in Equation (11). For high energy X-ray tube voltages, such as in RQA 9 of Figure 8b, the upper detector layer revealed $Q_{U}\left(0\right)=45.1\%$. The lower detector layer had a lower $Q_{L}\left(0\right)=25.5\%$ than the upper layer case because the remaining photons were utilized in the lower layer. Using the DFD, we can increase the DQE value to Q(0)=70.6% at the zero frequency. Note that this increase in DQE corresponds to a reduction in radiation exposure of more than 30%.

### 3.2. Registration Example of the Chest X-Ray Images

**Figure 9:**
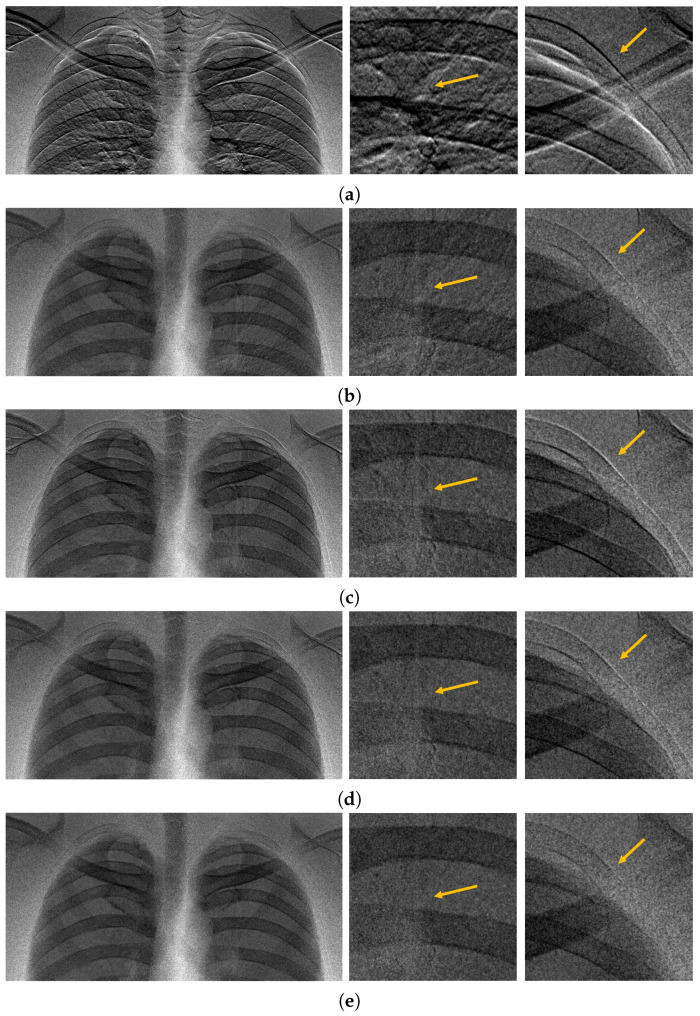
Example of chest-image registrations for a DFD. An image ratio of the upper and lower images is used to clearly observe the misalignment artifact (yellow arrows). (**a**) Image ratio without registration. Severe misalignments between the images are observed. (**b**) An intensity-based registration example from a conventional gradient-based method with least squares. The bronchioles are observed because of misalignment inside the lung. (**c**) Feature-based registration example (Harris). Misalignment is observed in the scapula area. (**d**) Image registration with the Fourier shift. A subpixel translation of (0.9582,6.8656) is estimated from the maximum-amplitude method [11]. Misalignment is observed toward the periphery of the image because of the projection. (**e**) Image registration from the proposed two-step method (Fourier shift and scale with cubic interpolation), where the scale factor is 1.0006875.

We now observe a registration example for the chest X-ray images acquired from the upper and lower layers, as in Figure 1. To observe misalignments between the images acquired from the DFD, we use the ratio of the upper and lower images and show several ratio images in Figure 9. We can observe from Figure 9a that the upper and lower images acquired from the DFD are severely misaligned. In Figure 9b,c, registration examples from conventional gradient-based and feature-based methods are shown. Here, a one-step transform with the translation and scale was conducted. The bronchioles inside the lung and lines outside of the lung are observed because of misalignment.

Based on the Fourier shift theorem, we can translate the lower image without attenuating its magnitude response. Here, the translation parameter was estimated based on the maximum-amplitude subpixel registration method. The registration result is shown in Figure 9d. However, because of the enlarged lower image caused by the X-ray projection, the alignment error increases as the pixel positions move further outward from the image. In the proposed registration method, the translated lower image is then scaled based on cubic interpolation, as shown in Figure 9e. We can observe very low misalignment errors over the entire image area.

## 4. Conclusions

In this paper, a two-step X-ray image registration method is proposed for a DFD. The first step is conducting a spatial translation according to the Fourier shift theorem using a spatial translation parameter estimated from a subpixel registration method based on the maximum amplitude. The second step is conducting a scale transformation using a cubic interpolation to process the X-ray projection. Conventional methods suffer from inconsistent registration accuracies depending on object shapes, pixel intensities, and interpolation methods in conducting a high-precision registration. However, the proposed two-step method showed better registration results than the conventional methods for a DFD with high precision.

## Figures and Tables

**Table 1:** Dual-layer flat-panel detectors (DFDs).

	Distance	CsI(Tl) Scintillator		Intermediate	TFT		
Detector	$d_{\mathrm{TT}}$	$d_{U}$	$d_{L}$	Filter	Photodiode	Stacking	Company
Lu et al. [6], 2019	2.5	0.2	0.55	1 Cu	a-Si	Normal	Varex Imaging, Salt Lake, UT, USA
Shi et al. [3], 2020	2.5	0.2	0.55	1 Cu	a-Si	Normal	Varex Imaging, Salt Lake, UT, USA
Kim [8], 2023	1.3–2.2	0.5	0.5	No filter, 0.5 Cu	a-Si/IGZO	Normal, inverted upper/lower	DRTECH, Seongnam, Republic of Korea
Wang et al. [5], 2023	-	0.2–0.55	0.55	No filter, 1 Cu	a-Si	Normal	Varex Imaging, Salt Lake, UT, USA
Su et al. [10], 2024	6.6	0.26	0.55	1 Cu	a-Si	Normal	CareRay, Suzhou, China
Lee & Kim [11], 2024	1.1	0.35–0.5	0.5	No filter	a-IGZO	Inverted lower	DRTECH, Seongnam, Republic of Korea

The unit of thickness is millimeter (mm).

## Data Availability

The original contributions presented in the study are included in the article, further inquiries can be directed to the corresponding author.

## Abbreviations

DFD	Dual-layer flat-panel detector
DQE	Detective quantum efficiency
MTF	Modulation transfer function
NNPS	Normalized noise power spectrum
NPS	Noise power spectrum
TFT	Thin-film transistor
SID	Source-to-image distance
